# Modulation of the neurotransmitter systems through the anti-inflammatory and antidepressant-like effects of squalene from *Aurantiochytrium* sp.

**DOI:** 10.1371/journal.pone.0218923

**Published:** 2019-06-28

**Authors:** Kazunori Sasaki, Mahmoud Ben Othman, Farhana Ferdousi, Masaki Yoshida, Makoto Watanabe, Kenichi Tominaga, Hiroko Isoda

**Affiliations:** 1 Alliance for Research on the Mediterranean and North Africa (ARENA), University of Tsukuba, Tsukuba, Ibaraki, Japan; 2 Interdisciplinary Research Center for Catalytic Chemistry, National Institute of Advanced Industrial Science and Technology (AIST), Tsukuba, Ibaraki, Japan; 3 Faculty of Pure and Applied Sciences, University of Tsukuba, Tsukuba, Ibaraki, Japan; 4 Algal Biomass and Energy System R&D Center (ABES), University of Tsukuba, Tsukuba, Ibaraki, Japan; 5 Faculty of Life and Environmental Sciences, University of Tsukuba, Tsukuba, Ibaraki, Japan; Radboud University Medical Centre, NETHERLANDS

## Abstract

Although algae have been the focal point of biofuel research, studies on their biological activities have been limited. In recent years, however, the importance of algae as sources of functional ingredients has been recognized due to their health beneficial effects. In this study, we evaluated the antidepressant-like activities of ethanol extract of *Aurantiochytrium* sp. (EEA) in the forced swimming test (FST)-induced depression in ICR mice. Imipramine, a commercially available tricyclic antidepressant drug, was used as positive control. Animals were administered EEA orally for 14 consecutive days and were subjected to the locomotor activity testing. Additionally, changes in gene expression in mice brain were assessed by real-time PCR and microarray assays to understand the molecular mechanisms underlying the effect of EEA. We found that the immobility time in FST was significantly reduced in the EEA-treated mice compared to that of in the control mice. Microarray and real-time PCR results revealed that EEA treatment induced changes in several genes in mice brain associated with pro-inflammation and dopaminergic, cholinergic, glutamatergic, and serotonergic synapses. It has previously been reported that several cytokines, such as IL-6 and TNF-α, which mediate neuroinflammation, are also responsible for indirectly altering brain neurotransmitter levels in neuropsychiatric disorders. Therefore, the regulation of the expression of pro-inflammatory genes in EEA-administered mice brain is considered to contribute to the enhancement of neurotransmitter systems-related gene expression in our study. Moreover, our *in vitro* study suggested that squalene, a component produced by *Aurantiochytrium*, was one of the active substances in EEA. In conclusion, our study provides the first evidence that *Aurantiochytrium* sp. can reduce neuroinflammation that may contribute to the modulation of the neurotransmitter systems, which could underlie its antistress and antidepressant effects.

## Introduction

Depression is a major cause of various psychiatric disorders worldwide. The World Health Organization predicts that depression will be one of the leading diseases by 2030 [1]. Depression is characterized by a wide range of symptoms, including low mood, loss of interest, significant weight loss, fatigue, malaise, lack of concentration, suicidal ideation, meaninglessness, excessive guilt, and difficulty in sleeping. The symptoms of depressive disorder cause significant clinical distress, dysfunction featuring anorexia, and most importantly impairment in social, occupational, or other areas of functioning [2].

Different classes of antidepressant drugs are currently available on the market, such as tricyclic antidepressants, selective serotonin reuptake inhibitors, serotonin-noradrenaline reuptake inhibitors, monoamine oxidase inhibitors, and noradrenergic and specific serotonergic antidepressants [3]. However, side effects of these drugs, such as nausea, headache, insomnia, excessive daytime sleepiness, sexual dysfunction, agitation, and weight loss or gain, are the problems for patients undergoing treatment for depression [1]. Therefore, it is considered to be an urgent matter to explore natural compounds as new remedies for depression with reduced side effects.

Falkowski and his colleagues have reported that microalgae were one of the earliest forms of life on the Earth that existed in Earth’s oceans more than 3 billion years ago, when the Earth’s environment was formed [4]. The vast diversity of microalgae (prokaryotic cyanobacteria and eukaryotic microbial algae) still remains largely unexplored [5–7]. It has been estimated that the number of microalgae species living in oceans and freshwater (lakes, ponds, rivers) ranges from 50,000 to 1 million. Presently, only 30,000 of these species have been studied [8]. Further, microalgae have been used by human as food for thousands of years [9]. In recent years, microalgal biomass has also gained increasing interest as an attractive source for the sustainable production of physiologically active substances, such as polyunsaturated fatty acids (PUFAs), carotenoids, phycobiliproteins, polysaccharides, and phycotoxins. We have recently reported the antidepressant-like effects of the colonial green alga *Botryococcus braunii* by modulating neurogenesis and enhancing dopaminergic function [10].

*Aurantiochytrium* is an oleaginous microorganism in the *Thraustochytriaceae* family that has attracted attention because of its ability to produce high levels of PUFAs and squalene. Recently, a research group of the University of Tsukuba isolated a novel strain of *Aurantiochytrium* sp. from the Okinawa prefecture in Japan, namely 18W-13a, which accumulates high amounts of squalene. The strain 18W-13a accumulated approximately 20% of squalene in glucose–peptone–yeast medium [11]. Squalene is a biosynthesized triterpene hydrocarbon and a precursor for all steroids in animals and plants. Squalene is used in the pharmaceutical and medical industry as it increases cellular and non-specific immune functions, decreases serum cholesterol levels, protects against gamma rays, and suppresses tumor proliferation [12–15]. Thus, these algae species have great potential as a renewable source of chemical products and as well as a new source for anti-depressant drugs. Moreover, to the best of our knowledge, there have been only few reports on the physiological effects of *Aurantiochytrium* sp. [16].

The objectives of this study were to evaluate the antidepressant-like effects of the ethanol extract of *Aurantiochytrium* sp. (EEA) using the forced swimming test (FST) in ICR mice and to further explore its possible molecular mechanism using DNA microarray analysis. We also focused our attention on changes in expression levels of genes associated with tumor necrosis factor-α (TNF-α), interleukin-6 (IL-6), and brain-derived neurotrophic factor (BDNF) in mice brain. In addition, the neuroprotective effects of EEA and squalene were investigated using human neuroblastoma SH-SY5Y cells.

## Materials and methods

### Preparation of EEA

The dried algal powder was extracted following previous report [10]. Dry powder of *Aurantiochytrium* sp. was provided by Algae Biomass and Energy System (ABES) R&D Center, University of Tsukuba, Japan. The dry powder was extracted in the dark using 99.5% ethanol, at room temperature for two weeks. The mixture was shaken at least once a day. Finally, the liquid fraction (EEA) was collected and filtered through a 0.22 μm filter (Merck Millipore, Billerica, MA, USA), and was used for *in vitro* assays. For the *in vivo* assay in ICR mice, EEA was concentrated using a SpeedVac (Thermo Fisher Scientific,) and the dried EEA was dissolved in milli-Q water.

### Preparation of squalene

Squalene was purchased from Wako Co, Ltd. (Tokyo, Japan). For the *in vitro* assays, Squalene was dissolved in the medium before further experiment.

### Animals

For the *in Vivo* studies, male ICR mice (8 weeks old) with average body weight of 35–40 g were purchased from Charles River, Japan. All mice were housed individually. Animals were provided with free access to food and water, except when subjected to EEA administration or testing. The animal house was maintained at a 12-h light/dark period, and the temperature was kept at 22 ± 1°C throughout the study. This animal experiment was approved by the Ethics Animal Care and Use Committee of the University of Tsukuba (16–042).

### EEA administration in ICR mice

After one week of acclimatization to the laboratory conditions, animals were randomly assigned into three groups (8 mice per group): control group, imipramine-administered group (20 mg/kg, daily), and EEA-administered group (100 mg/kg, daily). In our previously reported study, we orally administered 100 mg/kg of *Botryococcus braunii* to ICR mice to evaluate its antidepressant-like effects in the mouse FST [10]. Therefore, in the present study, we used a similar concentration of EEA for oral administration. EEA was dissolved in drinking water and was administrated by oral gavages in each mice of the treatment group for 14 consecutive days. The control group was administered an equivalent volume of tap water.

In our study, imipramine (a serotonin and noradrenaline reuptake inhibitor; SNRI) was used as positive control. It was dissolved in distilled water and was orally administered to mice at a volume of 20 mg/kg body weight for 14 days, as reported in our previous study [10].

### Forced swimming test

FST is a widely adopted behavioral animal model to investigate depression [17]. The FST was performed according to our two previous studies [10, 18]. To carry out the FST, the mice were placed individually in a cylindrical container having diameter 14 cm and height 25 cm. The container was filled with water (23 ± 1°C) up to 19 cm from the bottom, which was marked on the tank to confirm that the volume of water remain consistent across mice. The FST was carried out on days 1, 2, 6, 10, and 14 during the period of EEA oral administration. Mice were allowed to swim freely for six minutes, and only the last four minutes of the test were analyzed. This is because most mice are very vigorous at the beginning of the FST, and the possible effects of the treatment can be masked during the first two minutes [19]. The mouse was considered immobile when it showed disparity and became motionless in the water. During the period of immobility, mice would only make movements that were necessary to keep their head above the water.

### RNA isolation from the limbic area of mouse brain

Following the last FST trial on day 14, each mouse was sacrificed by cervical dislocation, and the whole brain was carefully isolated. The entire limbic area (100 mg) containing the cortex, hippocampus and amygdale was quickly dissected from mouse whole brain and washed with an ice-cold phosphate-buffered solution (PBS). The total RNA was purified using the ISOGEN kit (Nippon Gene Co. Ltd., Toyama, Japan) following the manufacturer’s instructions. The quantity and quality of total RNA was assessed with the NanoDrop 2000 spectrophotometer (Thermo Scientific, Wilmington, DE, USA).

### DNA microarray analysis

DNA microarray analysis was conducted on isolated RNAs extracted from the limbic area of mice brains as reported previously [10]. Double-stranded cDNA was synthesized from 100 ng of total RNA with the GeneAtlas 3´ IVT Express Kit (Affymetrix Inc., Santa Clara, CA, USA). Biotin-labeled amplified RNA (aRNA) was synthesized by *in vitro* transcription using the GeneChip 3´ IVT Express Kit (Affymetrix Inc., Santa Clara, CA, USA). Briefly, purified aRNA was fragmented using the GeneAtlas 3´ IVT Express Kit and hybridized for 16 h at 45°C using the GeneChip MG-430 PM microarray (Affymetrix Inc., Santa Clara, CA, USA). The chip was washed and stained in the Gene Atlas Fluidics Station 400 (Affymetrix Inc., Santa Clara, CA, USA), and the resulting image was scanned using the GeneAtlas Imaging Station (Affymetrix Inc., Santa Clara, CA, USA). Data analysis was performed using the Affymetrix Expression Console Software version and Visualization and Integrated Discovery (DAVID) software version 6.8 (National Institute of Allergy and Infectious Diseases (NIAID). Compared with the control (vehicle-treated group), fold-changes in the expression of genes in the imipramine- or EEA-treated groups were calculated and converted to linear data.

### TaqMan quantitative RT-PCR analysis of gene expression in the limbic area of mouse brain

On the basis of the results obtained from the microarray analysis, reverse transcription reactions were carried out with the SuperScript III Reverse Transcriptase (RT) kit (Invitrogen, Carlsbad, CA, USA). According to the manufacturer’s instructions, 1 μg of total RNA and 1 μL of oligo (dT) _12–18_ primers were denatured at 65°C for 5 min and were subsequently chilled at 4°C. After addition of SuperScript III RT (200 U), the reaction mix was incubated at 42°C for 60 min, followed by another 10 min at 70°C. All primer sets and TaqMan probes for experimental genes were purchased from Applied Biosystems (Foster City, CA, USA): mouse tumor necrosis factor-α (TNF-α) (Mm00447557_m1), mouse interleukin-6 (IL-6) (Mm00500992_m1), mouse brain-derived neurotrophic factor (BDNF) (Mm04230607_s1), and mouse GAPDH (Mm99999915_g1). For the mRNA quantification, TaqMan real-time PCR amplification reactions were carried out using an AB 7500 Fast Real-Time PCR system (Applied Biosystems). Amplifications were performed in 20 μL final volume, using 10 μL TaqMan Universal PCR Master Mix UNG (Applied Biosystems), 1 μL of the corresponding primer/probe mix, and 9 μL of template cDNA (final concentration 100 ng/20 μL). Cycling conditions were as follows: 2 min at 50°C, 10 min at 95°C, and 40 cycles at 95°C for 15 s followed by 60°C for 1 min.

### Cell culture

Human neuroblastoma SH-SY5Y cell line was obtained from American Type Culture Collection (ATCC). Cells were maintained in defined medium (DM) composed of Dulbecco´s modified Eagle´s medium/F12 medium (1:1 vol/vol) (Gibco, Japan) supplemented with 15% heat-inactivated fetal bovine serum (Bio-West, U.S.A) and 1% penicillin (5000 μg/mL)-streptomycin (5000 IU/mL) (PS) (Lonza, Japan) at 37°C in a 95% humidified air/5% CO_2_ incubator. A serum-free Eagle’s minimum essential medium (OPTI-MEM; Gibco, Japan) was used to culture the cells for the cell viability assay. The EEA used contained 20 mg/mL for *in vitro* assays.

### MTT assay

Cell viability was measured using the 3-(4,5-dimethylthiazol-2-yl)-2,5- diphenyltetrazolium bromide (MTT) method. SH-SY5Y cells (2 × 10^5^ cells·ml^−1^) cultured in 96-well plate (fibronectin-coated plate) (BD BioCoat, U.S.A.) were treated with EEA (1, 10, and 20 μg/mL) or squalene (1, 10, and 20 μg/mL) and subsequently with 500 μM dexamethasone (DEX, Wako, Japan) for 48 h. After sample treatment, 100 μL of Opti-MEM and 10 μL of MTT (5 mg/mL) were added, and the cells were incubated further for 6 h. The MTT formazan formed was dissolved in 100 μL of 10% SDS (w/v), and the absorbance was measured using a micro titer plate reader (Dainippon Sumitomo Pharma Co., Ltd., Japan).

### Statistical analysis

Results are expressed as mean ± standard error of the mean (SEM). Statistical analysis of the results obtained in the FST was carried out using two-way ANOVA followed by Ryan-Einot-Gabriel-Welsch multiple range test. A one-way within subjects ANOVA (repeated measures) followed by the Ryan-Einot-Gabriel-Welsch multiple range test was also carried out. The statistical evaluation was performed using the Student’s t-test between control and corticosterone-treatment groups in the *in vitro* experiment. A P value < 0.05 was considered statistically significant.

## Results

### EEA reverses the depression-like behavior in ICR mice induced by FST

**Fig 1:**
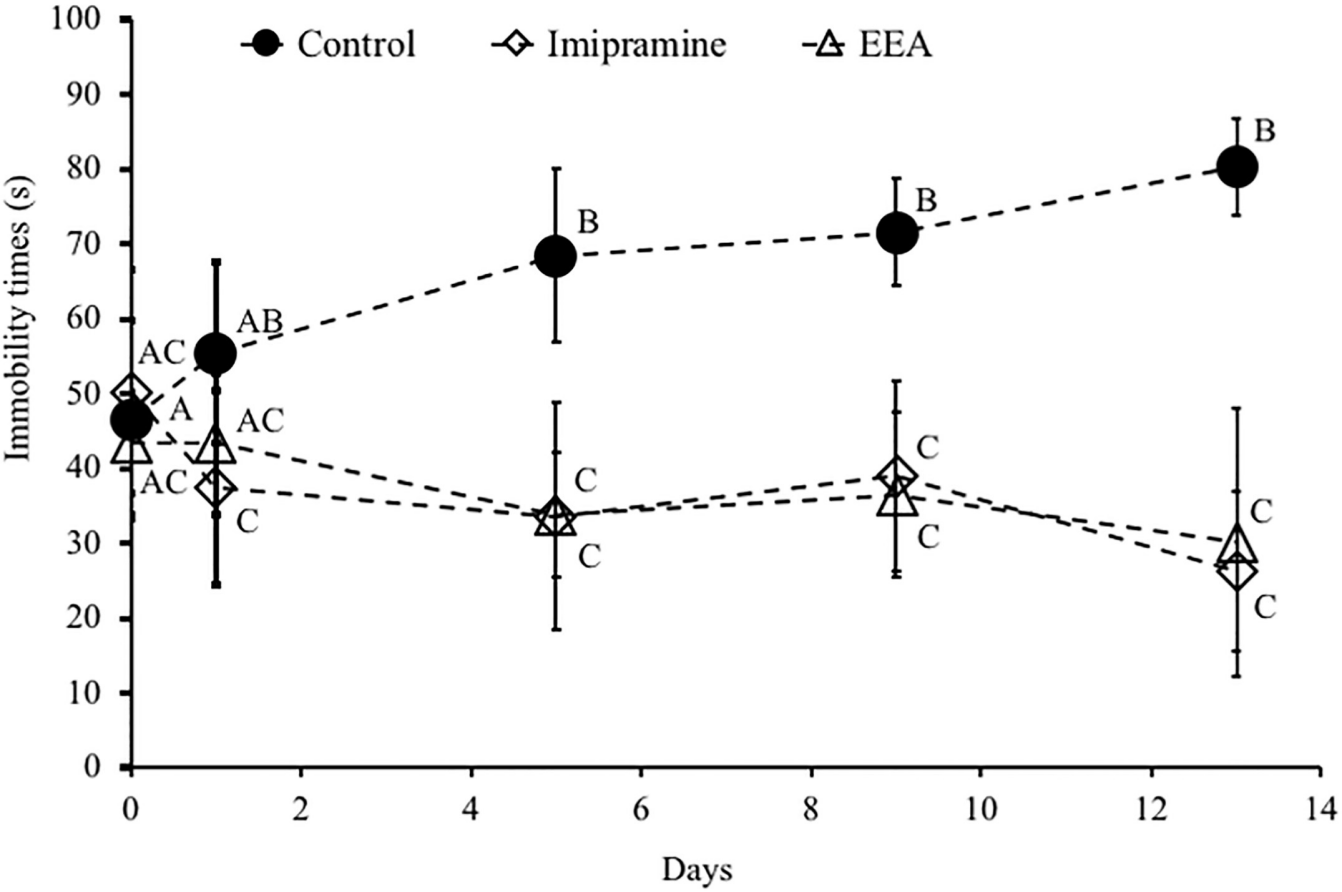
Effects of administration of EEA on the immobility time in the FST. Mice were orally administered daily with water (control), imipramine (20 mg/kg), or EEA (100 mg/kg) for 14 consecutive days. FST was carried out on day 1, 2, 6, 10, and 14. The immobility time during the final 4 min of a 6-min total session was measured. Data represent the mean ± SEM (n = 8). Values with different letters are significantly different at each measured time point (P < 0.05).

To determine whether EEA has antidepressant-like activity, its effect on FST-induced stress in mice was investigated. No death or sign of toxicity, such as significant loss of body weight, was observed in all the groups of mice (data not shown). As shown in Fig 1, the immobility time in the vehicle-treated control group gradually increased from the first session (or trial) to the 5th (D = day; D1, 46.5 ± 13.2 s; D2, 55.4 ± 12.0 s; D6, 68.4 ± 11.5 s; D10, 71.5 ± 7.1 s; D14, 80.3 ± 6.4 s, respectively) (Fig 1). However, this trend was not observed in the EEA-administered groups.

On day 14, the average immobility time for the EEA-administered groups (30.2 ± 18.0 s) was similar to the average immobility time measured in the imipramine-administered group (26.3 ± 10.7 s), which represented our positive control. In mice, treatment with both imipramine and EEA induced a 3-fold reduction of the average immobility time compared to the vehicle-administered control mice (80.3 ± 6.4 s).

### EEA-induced variations in genes involved in inflammatory signaling and dopaminergic-, glutamatergic-, cholinergic-, serotonergic pathway

To evaluate the molecular mechanism of the antidepressant-like effect of EEA, we investigated the changes in gene expression in the limbic area of the ICR mouse brain using microarray analysis. We found that the expressions of 28 genes were altered in the ICR mice administered with EEA compared to the control group (Table 1). Specifically, the expressions of protein kinase C, delta (*Prkcd*), adenylate cyclase 7 (*Adcy7*), phospholipase C, beta 4 (*Plcb4*), and son of sevenless homolog 1 (*Sos1*) genes associated with chemokine signaling pathway were downregulated in the EEA-administered groups. Moreover, the expressions of the following genes related to dopaminergic synapse were upregulated: protein kinase C, alpha (*Prkca*), adenylate cyclase 5 (*Adcy5*), inositol 1,4,5-trisphosphate receptor 1 (*Itpr1*), guanine nucleotide binding protein (G protein), gamma 7 (*Gng7*), adenylate cyclase 9 (*Adcy9*), glutamate receptor, ionotropic, AMPA2 (alpha 2) (*Gria2*), protein phosphatase 3, catalytic subunit, alpha isoform (*Ppp3ca*), calcium/calmodulin-dependent protein kinase II alpha (*Camk2a*), transient receptor potential cation channel, subfamily C, member 1 (*Trpc1*), dopamine receptor D1 (*Drd1*), dopamine receptor D2 (*Drd2*), guanine nucleotide binding protein, alpha stimulating, olfactory type (*Gnal*), and protein phosphatase 1, regulatory (inhibitor) subunit 1B (*Ppp1r1b*). In addition, the expressions of 10 genes associated with glutamatergic synapses were upregulated: *Prkcd*, *Adcy5*, *Itpr1*, *Gng7*, *Adcy9*, *Gria2*, *Ppp3ca*, glutamate receptor, metabotropic 3 (*Grm3*), glutamate receptor, ionotropic, kainate 3 (*Grik3*), and homer homolog 1 (*Homer1*). Further, we found the upregulation of 10 genes related to the cholinergic synapse: *Prkcd*, *Adcy5*, *Itpr1*, *Gng7*, *Adcy9*, *Camk2a*, Janus kinase 2 (*Jak2*), calcium/calmodulin-dependent protein kinase IV (*Camk4*), potassium voltage-gated channel, subfamily Q, member 5 (*Kcnq5*). Finally, the expressions of serotonergic synapse-related genes, *e*.*g*. *Prkcd*, *Adcy5*, *Itpr1*, *Gng7*, *Kras*, *Trpc1*, Rap guanine nucleotide exchange factor (GEF) 3 (*Rapgef3*), 5-hydroxytryptamine (serotonin) receptor 1B (*Htr1b*), and prostaglandin-endoperoxide synthase 2 (*Ptgs2*), were upregulated.

**Table 1:** Classification of differentially expressed gene names and their fold changes in the imipramine- and EEA-administered ICR mice in comparison to the control mice, as identified by DNA microarray analysis.

Gene Title	Gene Symbol	Imipramine vs Control	EEA vs Control	Related signaling pathways
adenylate cyclase 7	Adcy7	1.18	0.65 **	Chemokine signaling pathway
phospholipase C, beta 4	Plcb4	0.86	0.68 **	
protein kinase C, delta	Prkcd	0.73 *	0.47 **	
son of sevenless homolog 1 (Drosophila)	Sos1	0.76	0.77 *	
adenylate cyclase 5	Adcy5	1.55 *	1.82 **	Dopaminergic synapse
adenylate cyclase 9	Adcy9	0.93	1.27 *	
calcium/calmodulin-dependent protein kinase II alpha	Camk2a	0.77	1.29 *	
dopamine receptor D1	Drd1	3.88 **	4.69 **	
dopamine receptor D2	Drd2	1.94 *	2.44 **	
glutamate receptor, ionotropic, AMPA2 (alpha 2)	Gria2	1.02	1.26 *	
guanine nucleotide binding protein (G protein), gamma 7	Gng7	2.35 **	3.24 **	
guanine nucleotide binding protein, alpha stimulating, olfactory type	Gnal	1.46 **	1.51 **	
inositol 1,4,5-trisphosphate receptor 1	Itpr1	1.85 **	1.55 **	
protein kinase C, alpha	Prkca	1.18	1.54 **	
protein phosphatase 1, regulatory (inhibitor) subunit 1B	Ppp1r1b	2.81 **	4.25 **	
protein phosphatase 3, catalytic subunit, alpha isoform	Ppp3ca	1.09	1.45 *	
transient receptor potential cation channel, subfamily C, member 1	Trpc1	1.02	1.31 *	
adenylate cyclase 5	Adcy5	1.55 *	1.82 **	Glutamatergicc synapse
adenylate cyclase 9	Adcy9	0.93	1.27 *	
glutamate receptor, ionotropic, AMPA2 (alpha 2)	Gria2	1.02	1.26 *	
glutamate receptor, ionotropic, kainate 3	Grik3	1.16	1.46 *	
glutamate receptor, metabotropic 3	Grm3	1.11	1.34 *	
guanine nucleotide binding protein (G protein), gamma 7	Gng7	2.35 **	3.24 **	
homer homolog 1 (Drosophila)	Homer1	1.22	1.95 **	
inositol 1,4,5-trisphosphate receptor 1	Itpr1	1.85 **	1.55 **	
protein kinase C, alpha	Prkca	1.18	1.54 **	
protein phosphatase 3, catalytic subunit, alpha isoform	Ppp3ca	1.09	1.45 *	
Janus kinase 2	Jak2	1.07	1.22 *	Cholinergic synapse
v-Ki-ras2 Kirsten rat sarcoma viral oncogene homolog	Kras	0.98	1.23 *	
adenylate cyclase 5	Adcy5	1.55 *	1.82 **	
adenylate cyclase 9	Adcy9	0.93	1.27 *	
calcium/calmodulin-dependent protein kinase II alpha	Camk2a	0.77	1.29 *	
calcium/calmodulin-dependent protein kinase IV	Camk4	1.76 **	1.8 **	
guanine nucleotide binding protein (G protein), gamma 7	Gng7	2.35 **	3.24 **	
inositol 1,4,5-trisphosphate receptor 1	Itpr1	1.85 **	1.55 **	
potassium voltage-gated channel, subfamily Q, member 5	Kcnq5	1.08	1.94 **	
protein kinase C, alpha	Prkca	1.18	1.54 **	
5-hydroxytryptamine (serotonin) receptor 1B	Htr1b	1.95 **	2.1 **	Serotonergic synapse
v-Ki-ras2 Kirsten rat sarcoma viral oncogene homolog	Kras	0.98	1.23 *	
Rap guanine nucleotide exchange factor (GEF) 3	Rapgef3	1.15	1.27 *	
adenylate cyclase 5	Adcy5	1.55 *	1.82 **	
guanine nucleotide binding protein (G protein), gamma 7	Gng7	2.35 **	3.24 **	
inositol 1,4,5-trisphosphate receptor 1	Itpr1	1.85 **	1.55 **	
prostaglandin-endoperoxide synthase 2	Ptgs2	1.05	2.36 **	
protein kinase C, alpha	Prkca	1.18	1.54 **	
transient receptor potential cation channel, subfamily C, member 1	Trpc1	1.02	1.31 *	

Table values are expressed as mean ± SEM (n = 3 independent experiments) for three mice in each group.

*P < 0.05;

**P < 0.01.

### EEA-induced downregulation of TNF-α and IL-6 gene expression and up-regulation of BDNF gene expression in the limbic area of mouse brain

**Fig 2:**
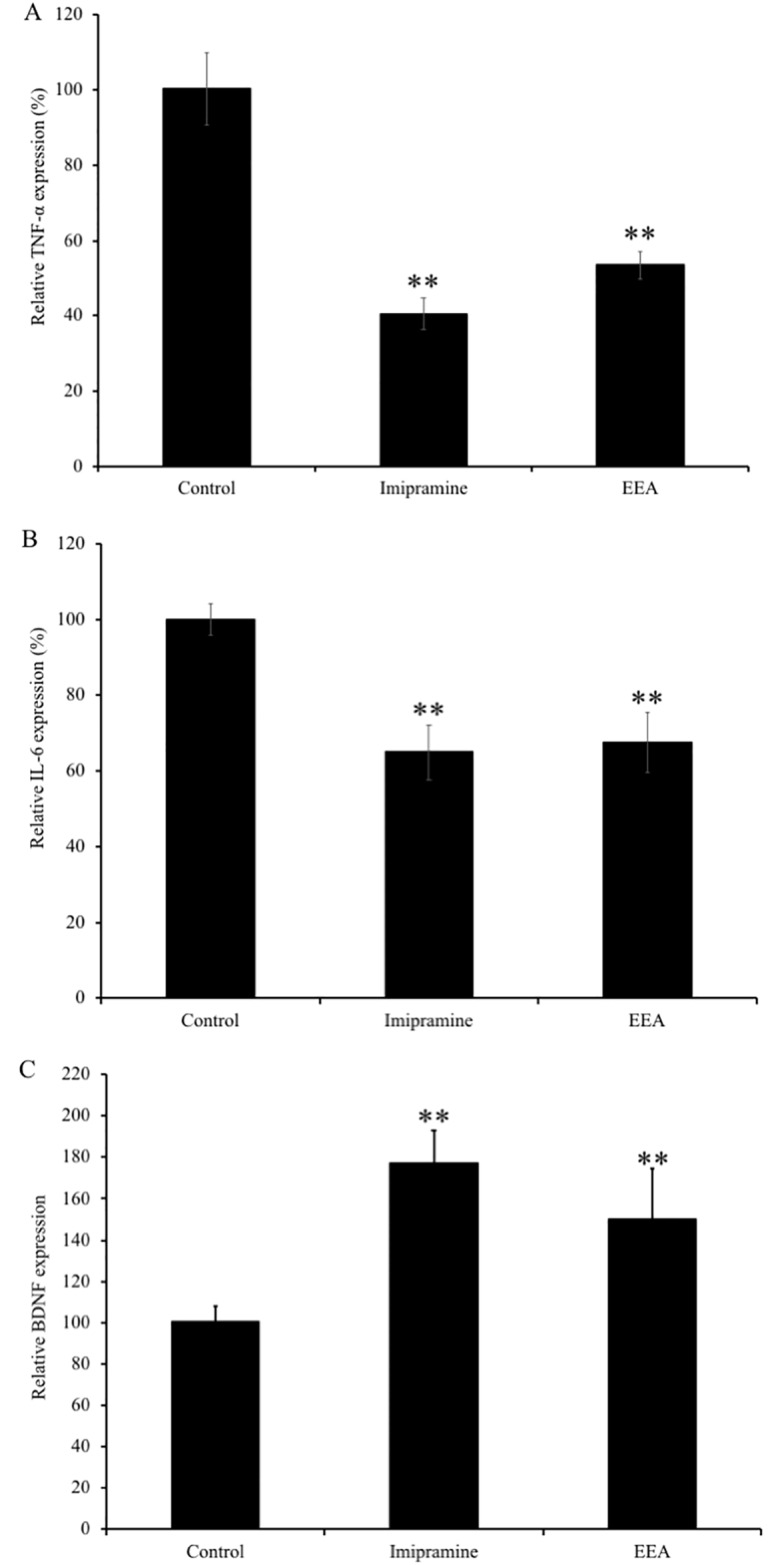
Effects of the administration of EEA on mRNA expression of PC, BDNF, and TH in the limbic area of ICR mice. Gene expression level of PC (A), BDNF (B), and TH (C) were normalized to GAPDH level and expressed as a ratio of the control group. Each bar represents the mean ± SEM (n = 5 independent experiments). * P < 0.05, ** P < 0.01 treatment vs. control group.

On the basis of the results obtained from the microarray analysis, we investigated the mRNA expression levels of major cytokines, TNF-α and IL-6, in the limbic area of ICR mouse of the four experimental groups. We also evaluated the mRNA expression levels of BDNF in ICR mouse brain as previous studies reported that cytokines interact with BDNF [20]. Our results showed that the mRNA expression levels of TNF-α were significantly downregulated in the imipramine- and EEA-administered groups (40.5 ± 4.3% and 53.4 ± 3.6%, respectively, compared to the control group; P < 0.01) (Fig 2A). Moreover, the mRNA expression of IL-6 was significantly downregulated in the imipramine- and EEA-administered groups (64.9 ± 7.4% and 67.5 ± 7.9%, respectively, compared to the control group; P < 0.01) (Fig 2B). Conversely, administration of imipramine and EEA induced overexpression of BDNF in mice brains (177.1 ± 18.2%, and 149.8 ± 24.1%, respectively, compared to the control group; P < 0.01) (Fig 2C).

### EEA- and squalene-treatment mediated protection from DEX-induced cell death

**Fig 3:**
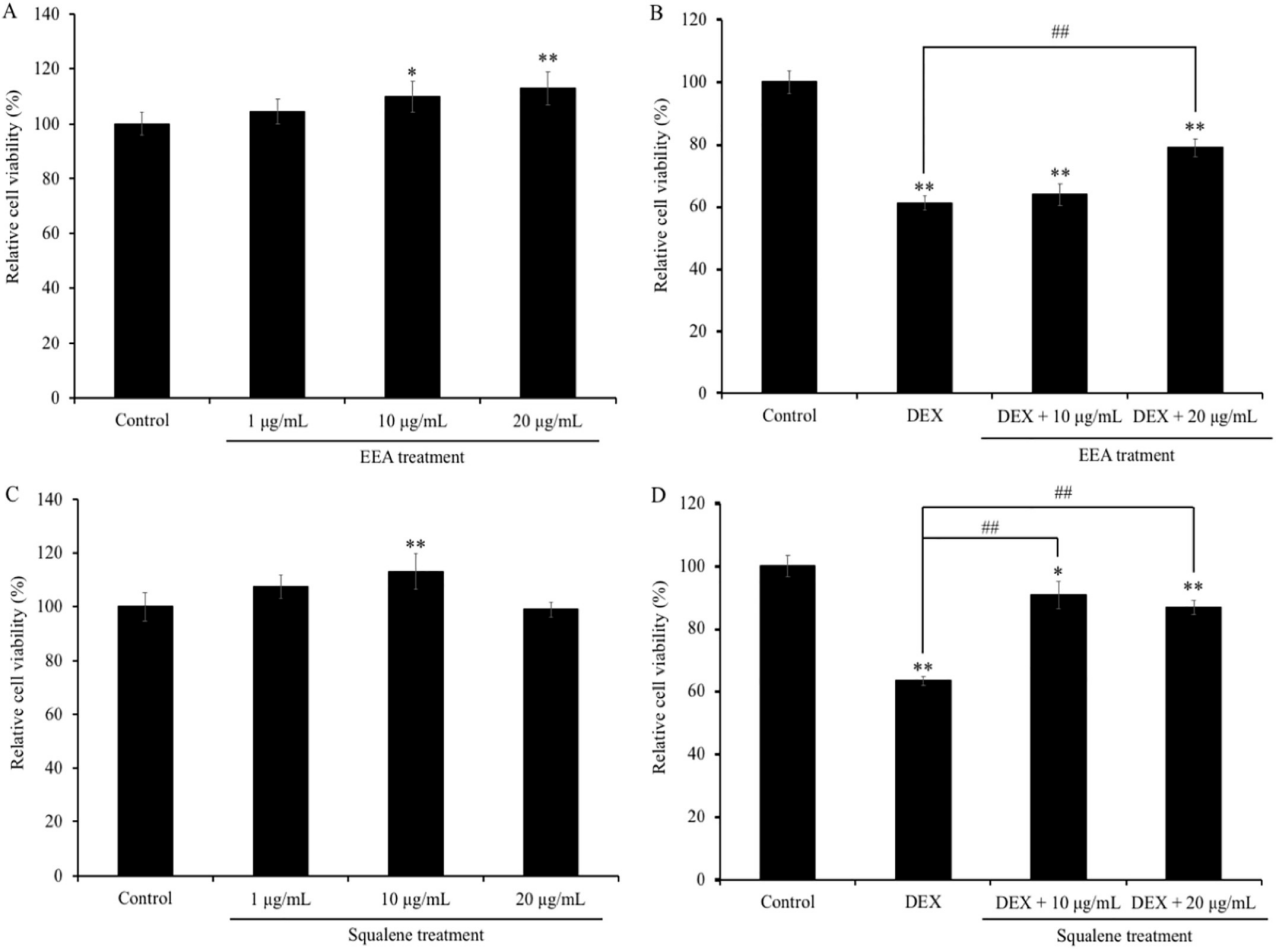
Effects of EEA (A) and squalene (C) on the cell viability and neuroprotective effects of EEA (B) and squalene (D) on the DEX-induced changes in SH-SY5Y cell viability. Each bar represents the mean ± SEM (n = 5). ** P < 0.01 vs. control cells, ## P < 0.01 vs. DEX-treated cells.

To evaluate the cytotoxicity of EEA and squalene, SH-SY5Y cells were treated with EEA (1, 10, 20 μg/mL) and squalene (1, 10, 20 μg/mL) for 48 hours, and cell viability was measured by the MTT assay. EEA and squalene showed no toxicity at all on cell viability (Fig 3A and 3C). Interestingly, EEA at the concentration of 20 μg/mL and squalene at the concentration of 10 μg/mL showed significantly increased cell viability up to 113.0 ± 6.1% and 113.3± 6.6%, respectively (P < 0.01) (Fig 3A and 3C). Further, MTT assay was carried out to evaluate the neuroprotective effects of EEA and squalene on SH-SY5Y cells pretreated with EEA (10 μg/mL or 20 μg/mL) and squalene (10 μg/mL or 20 μg/mL) for 10 min followed by DEX treatment (500 μM) for 48 hours; subsequently. The DEX-treated group showed a significant reduction in cell viability compared to the non-treated group. In contrast, pretreatment with 20 μg/mL of EEA ameliorated DEX-induced cytotoxicity up to 129.0% compared to DEX-treated cells (P < 0.01) (Fig 3B). Similarly, pretreatment with 10 μg/mL of squalene significantly increased cell viability up to 143.3% compared to the DEX-treated group (P < 0.01) (Fig 3D).

## Discussion

Depression is a complex mood disorder and its heterogeneity suggests that various biological mechanisms underlie the clinical presentations of depression. As far as we know, there is no scientific report on the antidepressant effects of *Aurantiochytrium*. In the present study, we evaluated the effect of *Aurantiochytrium* in animal models of FST-induced depression. The FST is widely used as a behavioral model to evaluate rodent depression in the screening of antidepressant drugs [17]. Specifically, the physical immobility of rodents in FST is thought to be an indication of behavioral despair or entrapment and is inferred as depressive-like behavior. Therefore, in the present study, we used FST to evaluate the antidepressive-like effect of EEA. In accordance with our previous studies [10, 18], we found prolonged immobility time was induced from the second FST experience in control ICR mice, suggesting depressive-like behavior in the present study. On the other hand, treatment with EEA resulted in a significant decrease in immobility time in the mouse FST. A similar response was observed in the imipramine-treated group; therefore, this result indicates that EEA might have antidepressant-like effects.

Recent researches have focused on *Aurantiochytrium* as a sustainable source of PUFAs, such as docosahexaenoic acid [21–23]. PUFAs have received great interest because of their health benefits and their widespread use in the food and pharmaceutical industries [24]. However, in the present study, we focused on the *Aurantiochytrium* sp. 18W-13a strain, which produces the highest level of useful hydrocarbon, squalene, compared with other algal strains [25]. We demonstrated that both EEA and squalene showed neuroprotective effects against DEX-induced neuronal cell death. Therefore, this result suggests that squalene is one of the active substances in EEA.

Neuroinflammation is a major contributing factor to a broad range of neuropsychiatric and neurodegenerative disorders. Chemokines are known to be important modulators of the immune response and activators of neuroinflammation [26] associated with psychiatric disorders, such as depression and anxiety disorders [27, 28]. In the present study, our results showed that several genes, such as *Adcy7*, *Plcb4*, *Prkcd*, and *Sos1*, related to chemokine signaling pathways were downregulated in EEA-treated mice brain. It has been reported that *Prkcd* may regulate four inflammatory chemokines, including *Ccl2*, *Mcp-1*, *Ccl7*, *Cxcl16*, and *Cx3cl1*. It was also reported that *Prkcd* stimulates *Ccl2* gene expression through the NF-B signaling [29]. *Sos1* may mediate *Cxcl12*-induced lymphocyte function-associated antigen 1 (*LFA-1*) activation, and *LFA-1* affinity triggering to *Cxcl12* is impaired by *Sos1* downregulation [30]. Therefore, downregulation of the expression of these chemokine-related genes is considered to contribute to the neuroinflammatory effect of EEA. In addition, previous clinical research confirmed that cytokines, namely TNF-α and IL-6, induce depressive mood, anxiety, impaired memory, and lack of concentration [31]. Moreover, Maes *et al*. reported in 1995 that depression is a disease caused by dysfunction of psychoneurotic immunity and activation of the inflammatory response system [32]. Because the etiology of depression is increasingly recognized as immune activation through secretion of proinflammatory cytokines, such as IL-1, IL-6, TNF-α, IFN-γ, leukotrienes, and prostaglandins, anti-neuroinflammatory activity has been proposed by many as a potential treatment for depression [33, 34]. Therefore, inhibition of proinflammatory mediators is also considered to be a key approach to control the progression of neurodegeneration and to alleviate the clinical presentation of psychiatric disorders. In the present study, real-time PCR results showed decreased gene expression of TNF-α and IL-6 in the mouse brain treated with EEA. Therefore, it can be postulated that EEA may have protective role against neuroinflammation via downregulation of genes associated with chemokine signaling and proinflammatory cytokines.

The monoamine neurotransmission system, which includes dopamine, serotonin (5-HT), and norepinephrine systems, has long been recognized as critically involved in the pathogenesis of depression. Studies reported that depression can be attributed to the functional imbalance or deficiency of monoamine neurotransmitters [35]. Further, accelerated production of inflammatory factors causes disruption of monoamine neurotransmitter metabolism, which lead to the neurological symptoms in psychiatric disorders. For example, the degeneration of dopaminergic neurons is an important characteristic of depression. The previous study has also reported that mild-to-moderate neuroinflammation can exacerbate the degeneration of dopaminergic neurons caused by a harmful stimulus [36]. Our microarray results show that EEA treatment upregulated several dopaminergic synapse signaling-associated genes in mice brain, such as *Adcy5*, *Adcy9*, *Camk2a*, *Drd1*, *Drd2*, *Gria2*, *Gng7*, *Gnal*, *Itpr1*, *Prkca*, *Ppp1r1b*, *Ppp3ca*, and *Trpc1*. Therefore, our study suggests that oral administration of EEA could enhance the dopamine pathway.

Moreover, the involvement of glutamatergic synapses in mood disorders was proposed on the basis of preclinical studies of NMDA receptor antagonists [37]. Several clinical studies have reported that glutamate levels were decreased in serum and cerebrospinal fluid of patients with mood disorders [38, 39]. Further, a recent study has revealed that proinflammatory cytokines, such as IL-6, lead to depletion of the TRP pathways and therby induce depression-like behavior and decrease glutamatergic activity [40]. We found that EEA administration increased the expression of glutamatergic synapse signaling-related genes, such as *Adcy5*, *Adcy9*, *Gria2*, *Grik3*, *Grm3*, *Gng7*, *Homer1*, *Itpr1*, *Prkca*, and *Ppp3ca*. Interestingly, the EEA-treated group showed a higher number of upregulated genes related to glutamatergic synapses compared to the imipramine-treated group. Therefore, EEA treatment is considered to activate glutamatergic synapses.

Additionally, we confirmed the upregulation of several genes related to cholinergic synapse signaling in EEA-treated mouse brains, such as *Jak2*, *Kras*, *Adcy5*, *Adcy9*, *Camk2a*, *Camk4*, *Gng7*, *Itpr1*, *Kcnq5*, and *Prkca*. Cholinergic neurons play a major role in the regulation of various CNS functions, such as excitation, attention, cognition, and memory. Impairment of cognitive function is often observed in major depressive disorders. Acetylcholine (Ach), restricted to cholinergic neurons, is detected at various sites outside of the central and peripheral nervous system, such as immune cells [41] and keratinocytes [42, 43] and has been reported to be associated with the immune system. Moreover, it was reported that ACh acts on α7 nAChR that is expressed on microglia and astrocytes and reduces neuroinflammation in the CNS [44, 45]. Therefore, dysfunction of cholinergic neurons can account for the onset of cognitive symptoms during the course of depression. Our present findings suggest that EEA may have effects on the improvement of the cholinergic synapse signaling dysfunction.

In addition, we showed upregulation of several genes related to serotonergic synapse signaling in EEA-treated mice brains, such as *Htr1b*, *Kras*, *Rapgef3*, *Adcy5*, *Gng7*, *Itpr1*, *Ptgs2*, *Prkca*, and *Trpc1*. Dysfunction of central serotonergic neurotransmission triggers the development of depressive symptoms. Serotonergic system is an important target of classical antidepressant drugs [46]. Previous research has also reported that Pioglitazone, a peroxisome proliferator-activated receptor gamma (PPAR-γ) agonist, has exhibited antidepressant-like effects through modulation of the NF-κB/IL-6/STAT3 and CREB/BDNF pathways and regulation of stress-induced expression of proteins involved in central serotonergic neurotransmission [47]. The results of the present study may suggest that EEA treatment could prevent serotonergic synapse signaling dysfunction.

Several studies support the role of reduced BDNF activity in inflammatory cytokine-associated depression [48, 49]. Thus, in addition to other mechanisms, a reduction in BDNF may ultimately be the reason for the development of depression due to stress-induced neuroinflammation. It has been reported that LPS-induced inflammation decreased BDNF in the hypothalamus, contributing to depression-like behavior [50]. Our evidence suggested that EEA treatment increased BDNF gene expression, contributing to the regulation of depression-like behaviors caused by neuroinflammation.

## Conclusions

Development of new strategies for the prevention and treatment of psychological diseases is a major therapeutic challenge. Our results showed that the significant upregulation of several genes involved in the neurotransmitter systems, such as dopaminergic and serotoninergic synapses, through the significant reduction of proinflammatory-related genes contributes to the antidepressant-like effect of EEA. Altogether, our results suggest that *Aurantiochytrium* may have therapeutic potential for the treatment of neuropsychiatric symptoms in neurodegenerative diseases.
